# P02 Horizontal transfer of antimicrobial resistance genes from *Aeromonas salmonicida* to *Escherichia coli* via conjugation

**DOI:** 10.1093/jacamr/dlaf118.009

**Published:** 2025-07-14

**Authors:** Surya Prasad Dahal, Hetron Mweemba Munang’Andu, Henning Sørum, Saurabh Dubey, Kiron Vishwanath, Bo Peng

**Affiliations:** Faculty of Biosciences and Aquaculture, Nord University, Bodø, Norway; Faculty of Biosciences and Aquaculture, Nord University, Bodø, Norway; Faculty of Veterinary Medicine, Department of Paraclinical Sciences, Norwegian University of Life Sciences, Ås, Norway; Faculty of Veterinary Medicine, Department of Paraclinical Sciences, Norwegian University of Life Sciences, Ås, Norway; Faculty of Biosciences and Aquaculture, Nord University, Bodø, Norway; Sun Yat-sen University, Guangzhou, China

## Abstract

**Background:**

Furunculosis, a bacterial infectious disease affecting salmonid fish species, is caused by the water-borne Gram-negative bacterium *Aeromonas salmonicida* subspecies *salmonicida* capable of hosting drug resistance plasmids carrying various antimicrobial resistance genes and genetic structures as integrons with ability to adapt the contents of the antibiotic resistance genes in the genome. The spread of antimicrobial resistance (AMR) genes among bacterial species poses a significant threat to both animal and public health.

**Objectives:**

In this study, we investigated the horizontal transfer of AMR genes from *Aeromonas salmonicida* to *Escherichia coli* (DH5α) through conjugation.

**Methods:**

Three strains of *A. salmonicida* (1711/93, 1995/91 and 2402/89) harbouring the resistance genes *sul1*, *tet*(A), *dfr16* and the class 1 integron on the transferable R plasmid pRAS1were used as donors, while *E. coli* DH5α, lacking antibiotic resistance genes and integrons, served as the recipient. Antimicrobial susceptibility testing using the disc diffusion method confirmed resistance in *A. salmonicida* to sulfamethoxazole, tetracycline and trimethoprim. Conjugation experiments were performed at room temperature for 24 h, and transconjugants were selected on Mueller–Hinton agar supplemented with the respective antibiotics.

**Results:**

Growth in the inhibition zones around sulfamethoxazole, tetracycline and trimethoprim discs indicated the acquisition of resistance by *E. coli*. PCR amplification and Sanger sequencing confirmed the presence of *sul1*, *tet*(A), *dfr16* and the integron in the transconjugants, demonstrating the successful transfer of pRAS1 with its AMR genes.

**Conclusions:**

This study highlights the role of conjugation in the dissemination of antibiotic resistance and underscores the potential for AMR gene transfer between environmental and clinically relevant bacteria, emphasizing the need for continued surveillance and control of AMR spread.

